# SAR and optical images correlation illuminates post-seismic landslide motion after the Mw 7.8 Gorkha earthquake (Nepal)

**DOI:** 10.1038/s41598-022-10016-2

**Published:** 2022-04-15

**Authors:** Pascal Lacroix, Théo Gavillon, Clément Bouchant, Jérôme Lavé, Jean-Louis Mugnier, Samir Dhungel, Flavien Vernier

**Affiliations:** 1 ISTerre, University Savoie Mont-Blanc, CNRS, IRD, University Grenoble Alpes, 38000 Grenoble, France; 2 The Njord Centre, Departments of Geosciences and Physics, University of Oslo, Blindern, box 1048, 0316 Oslo, Norway; 3 Centre de Recherches Petrographiques et Geochimiques, CNRS, Université de Lorraine, 54501 Vandoeuvre-les-Nancy, France; 4 Tribhuvan University, Kirtipur, 44618 Nepal; 5 LISTIC, University Savoie Mont-Blanc, Annecy, France

**Keywords:** Natural hazards, Seismology, Hydrology

## Abstract

In the days to weeks following an earthquake, landslides can display specific post-seismic motions, including delayed initiations and post-seismic relaxations. These motions have an uncertain origin, sometimes attributed to specificities of the landslide basal interface or to fluid transports in the landslide basal shear zone. Here we address this question, by documenting the co- and post-seismic motions of slow-moving landslides accelerated by the Gorkha earthquake (Mw 7.8, 25/04/2015, Nepal). We detect 11 slow-moving landslides over an area of 750 km$^2$ in the near field of the earthquake, and monitor their motions thanks to a time-series of Pléiades optical satellite images and SAR Sentinel-1 images. The post-seismic landslide motions are much larger than the co-seismic ones, reaching up to $34 \,\pm\, 0.6$ m accommodated over 2 months. A delayed initiation of several days (> 4 days) is also measured for at least two of the landslides. We analyze our findings in regards with all the previous observations on slow-moving landslides accelerated by earthquakes, and propose that the post-seismic motions are caused by diffusion of groundwater from co-seismic material contraction up to the landslide basal shear zone or from internal landslide reconfiguration. Our observations strongly suggest the main control of the hydrology in the landslide processes under seismic forcings.

## Introduction

Landslides are the main secondary effects of earthquakes in mountainous environments, often claiming more than a third of the casualties during the shaking^1^. Both the dynamic loading^2^ and grain crushing at the landslide basal surface^3^ can explain the co-seismic triggering of numerous landslides. In addition, different studies also report post-seismic activity of landslides, including (i) delayed landslide triggering after earthquakes from several hours to several days^4^, (ii) post-seismic accelerations of slow-moving landslides^5^, and (iii) increased landslide rates in the months to years following the main shaking^6–8^. These observations are not consistent with a landslide triggering caused by the dynamic loading or grain crushing at the interface, and show that the risk associated with earthquake-triggered-landslides is not limited to the brief moment around the earthquake shaking, but can last for years.

To infer the complex mechanics of post-seismic landslide movements, multi-parameter monitoring of slow-moving landslides^9^ dynamics in seismic environments has been undertaken^5,10,11^. These studies show a co- and a post-seismic acceleration of the landslides over several weeks, and material damage (i.e. cracking or changes of the void space in the bulk) over several months. This bulk damage is found to ease the water infiltration during the subsequent rainfall events over the months after major earthquakes^11^, and can therefore explain the increased landslide rates observed at the regional scales in the months to years after the earthquake^7^. However, the physical processes of the post-seismic motion of landslides in the weeks following the shaking, observed on different slow-moving landslides^4,5,11–13^, even without precipitation, are still uncertain.

**Figure 1:**
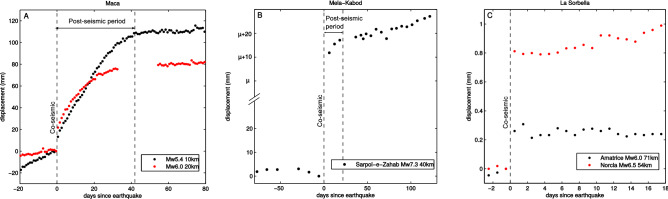
Time-series of slow-moving landslide displacements in the days around several earthquakes. (**A**) GNSS time-series on the Maca landslide (Peru) during 2 local earthquakes (adapted from^5^ and^11^), (**B**) InSAR time-series on the Mela-Kabod landslide (Iran) during a Mw 7.3 earthquake (adapted from^13^, $\mu$ represents a co-seismic motion of about 34 m calculated with a satellite image correlation), (**C**) Inclinometer time-series on La Sorbella landslide (Italy) during 2 distant earthquakes (adapted from^10^). No rainfalls were recorded in the time-periods shown.

The few measurements of slow-moving landslide kinematics during earthquakes, conducted over landslides of different lithologies and mechanisms (see Table S3), show very different co- and post-seismic behaviours at weekly/monthly scales. First, these two components have a large variability of magnitudes. The Maca (Peru) and Mela-Kabod (Iran) landslides (Fig. 1A and B) show a post-seismic transient motion of between 5 mm and 80 mm. La Sorbella (Italy) landslide does not (Fig. 1C). A landslide situated in the far-field of the Sarpol-e-Zahab earthquake (Mw 7.3, Iran) does not display a post-seismic transient motion^13^, whereas the Mela-Kabod landslide, situated in the near field of the same earthquake, displays at least 5 mm of post-seismic motion in the 3 weeks following the shaking (Fig. 1B). The ratio between co- and post-seismic motions varies a lot from one case study to the other, from no post-seismic motion^10^ to post-seismic motions three times larger than co-seismic ones^5^. Furthermore, two earthquakes do not produce similar co- and post-seismic motion ratios on the same landslide. On the Maca landslide (Fig. 1A), for instance, a Mw 5.4 earthquake situated at 10 km produced a smaller co-seismic motion than a Mw 6.0 earthquake situated at 20 km, but a greater post-seismic motion. This anti-correlated behaviour suggests that the co- and post-seismic motions have a different origin. Based on these observations, the landslide post-seismic transient motion can not be directly attributed to the seismic stress perturbation on the landslide shear zone.

Different hypothesis have been proposed to explain the mechanics of the post-seismic behavior, including transient changes in groundwater levels^14^, and/or damage of the bulk landslide material^11^, and/or specific properties of the basal frictions^5^. Rate-and-state friction laws of the landslide basal interface fit well the Maca landslide transient post-seismic motion^5^, but the results lead to parameters *a* and *b* of the model far much smaller than previous estimates obtained on active faults ($\sim$10$^{-15}$ against $\sim$10$^{-2}$). These low values suggest either that the contact at the sliding surface is essentially supported by the elastic contacts (which can be explained, for instance, by an applied normal stress much lower for landslides than for tectonic faults or lab-scale experiments)^15^, or that the rate-and-state friction agreement with the Maca landslide dynamics was accidental.

Other hypothesis related to effects of fluid migration during the shaking^16^, or variations of the material permeability^17^ must also be investigated. However, the observation of transient post-seismic motions of landslides is still limited to a few number of case-studies^5,10,11,13^ in the intermediate and far field of earthquakes (distance at least 2-3 times the fault length), where seismic shaking has limited groundwater effects^18^. All these uncertain conclusions show the need for more observations of post-seismic landslide motions to better infer the underpinning mechanisms.

In this study, in order to understand the mechanisms of this post-seismic motion at a weekly/monthly scale, we document the kinematics of slow-moving landslides accelerated by the Gorkha earthquake (Mw 7.8, 25 April 2015, Nepal). We provide unique observations of landslide kinematics in the near field of an earthquake that we analyze in regards with previous observations.

## Study site

**Figure 2:**
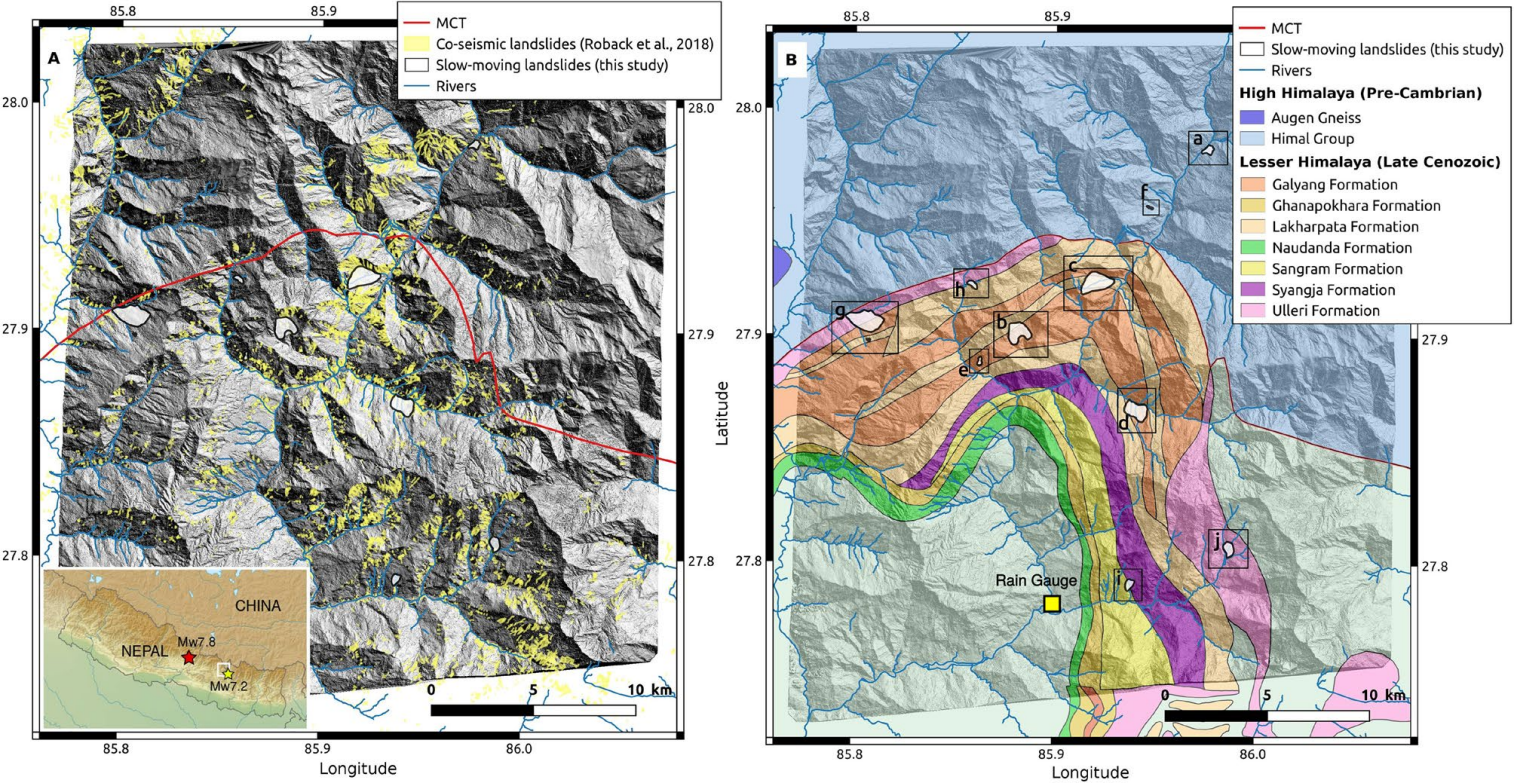
Topographic (**A**) and geological map (**B**) of the site study in Nepal. The hillshade topography is the DEM produced using Ames Stereo Pipeline v2.5.3 on the Pléiades stereo acquisitions from December 2015 (see “Methods”). The white shapes with black contours correspond to the slow-moving landslides detected in this study. The Main Central Thrust (MCT) is represented with a thick red line. On panel (**A**) the co-seismic landslide inventory is based on^21^. On panel (**B**), the black frames correspond to the zooms around the slow-moving landslides shown in the Supplementary materials (noted from a to j). The geological map is based on 1/250000 quadrangle maps of the Department of Mines and Geology. The inset shows the locations of the main shock of April 25 2015 and its aftershock of Mw 7.3 on 12 May 2015, close by the area of study. The rain gauge of Bahrabise is shown with a yellow square. QGIS^23^ was used to create this figure.

The Gorkha earthquake of Mw 7.8 (25 April 2015) struck the central part of Nepal (Fig. 2). The main shock nucleated on the western part and ruptured a segment of the Main Himalayan Thrust of 150 km long at 15 km depth with an eastward directivity^19^. The main shock was followed on 12 May 2015 by an aftershock (Dolakha earthquake) of Mw 7.3 on the eastern side of the main rupture. The seismic sequence triggered at least 25000 landslides^20,21^. These landslides are mainly attributed to a co-seismic triggering due to the dynamic loading of the steep slopes of the high Himalayas in fractured rockmass^22^ and weakened regolith^21^.

At least 90% of the landslides occurred before the Dolakha earthquake^21^, but the exact timing of the landslide occurrence is biased by the detection methodology, which is function of the optical satellite image acquisitions and cloud coverage. Many of the landslides, including the large and destructive ones in Langtang valley north of Kathmandu^24^ were co-seismic. For many others, the exact timing remains uncertain due to cloud coverage preventing the landslide detection on a daily basis from satellite optical images. A few ones, like the Baisari landslide^25^, 100 km at the West of the main rupture, have occurred up to one month later. Landslide inventories conducted in the 3 years after the earthquake, show that the post-seismic landslide activity has remained high, and that the areas affected in a post-seismic manner are different that the ones affected co-seismically^26^.

In contrast to the catastrophic impact of the Gorkha eathquake on disrupted landslides triggering, which has been widely documented, very few studies have focused on its impact on slow-moving landslides and their possible reactivation or acceleration. The central Himalaya, however, has been described as hosting many dormant or slow-moving landslides^22,27–29^. Along the Trisuli valley, NW of Kathmandu, slow deformations (tens of mm per year) of 6 deep seated landslides before and after the Gorkha earthquake have been evidenced using InSAR techniques^29^. However, because of high noise during the monsoons, the behaviour during the co-seismic phase and the months that followed it can not be documented. Therefore, in this study, we propose to detect and monitor slow-moving landslides affected by the seismic sequence and the subsequent monsoons from image correlation techniques, in an area characterized by a high density of co-seismic disrupted landslides, evidences of shattered ridge^30^ and the previous description of numerous dormant large landslides^28^.

Our area of interest is chosen around the Bhote Koshi valley, at the termination of the Gorkha earthquake rupture and in the area affected by the Dolakha earthquake (Fig. 2). This region is one of the most affected by the co-seismic landslides^21^, and is struck by intense monsoons of more than 2.5 m per year, as estimated by the monthly rainfall GPCC-v2018 dataset^31^ or measured on a daily basis by a rain-gauge of the DHM (Department of Hydrology and Meterology of Nepal) located at Bahrabise, $\sim$10 km south of the study area (Figs. 2 and S8). The area of study is characterized by steep slopes along the Bhote Koshi valley. Geologically, the Main Central Thrust (MCT) zone runs through this area (Fig. 2). It corresponds to a major tectonic feature, associated to intense ductile and brittle deformations and marks the transition between the High and Lesser Himalaya lithologic Units. The High Himalaya is mostly made up of high grade para- and orthogneisses. The Lesser Himalayan lithologies, in particular the upper units below the MCT zone, are composed of alternations (tectonic stacking) of micaschists, slates, carbonaceous phyllites, limestones, dolomites, quartzites and orthogneisses (Fig. 2B).

## Results

In this study, the strategy developed to detect and monitor slow-moving landslides over an area of 750 km$^2$ relies on the measurements of ground motion thanks to image correlation techniques applied on two types of satellite images (Fig. S1 and Table S1): (1) 6 optical images from Pléiades satellites, acquired between June 2014 and December 2017, that present the advantage of a very high spatial resolution of 0.7 m and therefore a good ability to detect landslides even of small sizes, (2) C-band radar images from Sentinel-1 satellite, that offers an acquisition every 24 days before the earthquake and 12 days after, even under cloud conditions. This satellite-based analysis is complemented by visits on the fields to provide a ground truth and characterization of the landslides detected.

### Satellite detection of landslides and field observations

**Figure 3:**
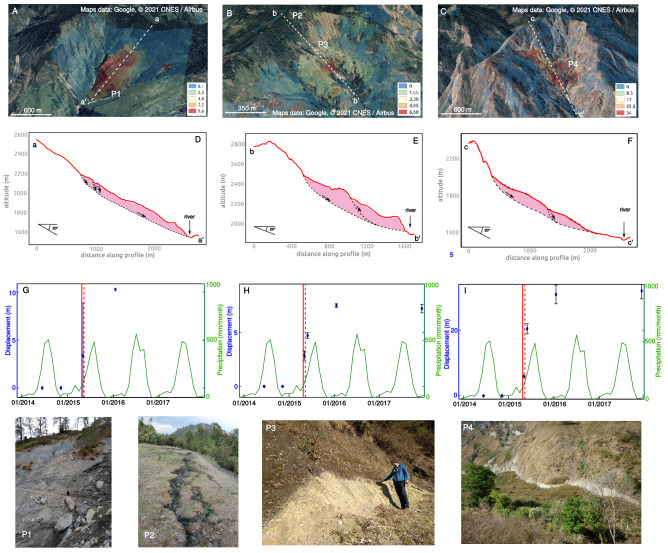
Pléiades displacement fields (see section Methods) overlayed over a Google-Earth view (**A**–**C**), interpretative cross-sections (**D**–**F**) , and time-series of the cumulative displacement obtained from Pléiades data (**G**–**I**) and monthly rainfall extracted from the GPCC-v2018 dataset^31^ on 3 detected landslides: Tapgaon (**A**,**D**,**G**), Gumba (**B**,**E**,**H**), and Duguna Gadi (**C**,**F**,**I**). Pictures show some key general features observed on these landslides: colluvial debris including very large blocks (20 × 20 × 20 m$^3$) both on the surface and outcropped by the river erosion (P1), shallow fractures on the above ridges (P2), head- (P3) and lateral- (P4) scarps reaching up to 30 m. Google-Earth (https://earth.google.com/) was used to create the subfigures (**A**–**C**). Inkscape (www.inkscape.org) was used to create subfigures (**D**–**F**). Matlab (www.mathworks.com) was used to create subfigures (**G**–**I**).

Using Pléiades imagery, we detected 11 slow-moving landslides (Table 1, Fig. 2 and zooms in the Fig. S5 of the Supplementary Materials). The landslides are almost all (9 over 11) situated in the Lesser Himalaya lithologic Units. The landslides, noted from a to j (see Fig. S5) for simplicity, have an active area between 0.025 and 1.3 km$^2$, much larger than the rapid landslides that affected the area, and mean altitudes varying between 1700 and 2300 m asl. Their maximum velocities during the year 2015 (Dec 2014–Dec 2015) estimated with the Pléiades data are between $2\, \pm \, 0.6$ and $34\, \pm\, 0.6$ m/yr with a median value of 6.5 m/yr. Their mean slopes (26.2 ± 5.3°, Table 1) are much lower than for co-seismic rapid landslides^20^ (50 ± 13°), showing a different type of landslide. We visited 8 of these landslides during two fieldtrips in May 2016 and January 2019. All the landslides exhibited clear signs of instability with fresh head- or lateral- scarp motions up to several meters (Fig. 3), and cracks on the landslide. Local testimonies were also collected, that confirmed the high activity of most of these landslides in 2015.

**Table 1:** Detected landslide characteristics (see their locations on Fig. 2 and the zooms on Fig. S3). *polygenic* = landslides and rockfall debris accumulation. *monogenic* = lower part of a former hillslope collapse (i.e. fragmented and weathered bedrock). ?= not visited on the field (the origin of the material is only estimated from topographic settings observed on Google Earth). $^*$ = probable limited displacements (slope of the slided mass not very different from the regional hillslope gradient). $^{\#}$ = probable large displacements (slope of the slided mass much shallower than the regional hillslope gradient).

Landslide (subfig)	Location (lon, lat)	Mean altitude (m)	Area (m$^2$)	Mean slope ($^o$)	Max 2015 velocity (m/yr)	Nature of landslide material
Zhangmu (a)	85.975°, 27.982°	2050	177,500	32.1	$2\,\pm \,0.6$	monogenic?
Gumba (b)	85.882°, 27.901°	2200	831,400	27.3	$6.5\,\pm\, 0.4$	polygenic
Duguna Gadi (c)	85.919°, 27.924°	1800	1,127,500	18.2	$34\,\pm\, 0.6$	polygenic
Pokhan (d)	85.938°, 27.868°	1700	773,000	25.9	$8\,\pm \,0.4$	monogenic?
Listikot (e)	85.863°, 27.888°	1900	78,900	31.5	$4.5\,\pm\, 0.6$	monogenic$^*$
Kodari (f)	85.946°, 27.957°	2000	24,600	28.0	$9.5\,\pm\, 0.5$	polygenic
Tapgaon (g)	85.805°, 27.907°	1900	1,293,400	20.2	$12\,\pm\, 0.5$	monogenic$^*$
Tapgaon-2 (g)	85.808°, 27.898°	2100	28,200	29.7	$11\,\pm\, 0.5$	monogenic
Chagam (h)	85.859°, 27.922°	2320	123,500	29.7	$4.5\,\pm\, 0.07$	polygenic
Karthali (i)	85.938°, 27.790°	1450	185,000	16.9	$3.5\,\pm\, 0.9$	monogenic
Dolangsa (j)	85.987°, 27.807°	2075	291,000	28.7	$4\,\pm\, 1.1$	monogenic?$^{\#}$

One of these landslides (Duguna Gadi, Fig. 3C and F), was previously described as an old rockslide deposit at the bottom of a small cirque-shaped valley dominated by steep rocky slopes^32^. Rapid landslides triggered by the earthquake sequence supplied the cirque with a significant amount of rock debris along its western side. This might suggest that the paleo-landslide mass is not monogenic but results from numerous episodes of debris filling supplied by the surrounding slopes. Whatever the origin of that mass, outcrops within eroded gullies display breccia material with a large variety of granulometry, with cobbles and boulders into a silty sands matrix. A major fresh scarp can be observed all along the northern side of the deposit, at the contact between the debris mass of the cirque and the steep rocky slopes (picture P4 on Fig. 3). Even if additionnal and less extended scarps are located within the paleo-landslide mass, this observation corroborates the Pléiades displacement fields and suggests that the whole breccia deposit is affected by the gravitational motion. Three other landslides (b, f and j) share similar features, i.e. reactivation of gently sloping paleo-landslide deposits in a cirque-shape upper valley. The Gumba (b) slide displays also a major and continuous head scarp at the contact between the brecciatic mass of debris and the rocky valley walls (Picture P3 on Fig. 3), whereas at the slides (f,j) only the bottom part of the debris deposits is affected by the motion according to the Pléiades image detection (Supplementary Materials).

The other identified activated landslides (a, d, e, g, h and i on Fig. 2 and Fig. S5, and Table 1) occur along more rectilinear valley sides on slopes ranging from 20 to 32°. They could correspond to the reactivation of a debris or colluvial cover, or to deeper structures like deep seated landslides or DSGSD (deep seated gravitationnal slope deformation) affecting fractured and weathered bedrock. At the Tapgaon landslide (g, Fig. 3A), the head- and lateral scarps are steep: these sub-vertical discontinuities of several meters (up to 15 m ), exposing deformed regolith and weathered bedrock, suggest a relatively deep rooting of the landslide. At the riverbed incision, the bedrock outcrops (Picture P1 on Fig. 3), and delimits the lateral boundary of the active zone as seen in the Pléiades displacement fields (g,j). The Pléiades images show a variety of displacement fields: fairly uniform displacement fields (a,c,e,g,h) suggesting translational slides, or velocity profiles made of blocks with higher velocities at the toe (b,d,i), suggesting rotational mechanisms. Sharp topographical discontinuities at the landslide toe, cut by deep rivers, suggest the main control of the river erosion in the landslide dynamics over the long term (a,b,c,d,g,i,j). In some cases (b,c,d,h), large fractures were found on the ridges above the deposits (Picture P2 on Fig. 3), showing the source of future landslides, feeding the paleo-landslide deposits. For all these reasons, we classify the landslides as either thick translational rockslides (g) or reactivations of very thick paleo-landslides deposits (a,b,c,d,i,j) with rotational or translational motions, or mobilization of shallow colluvial debris cover (e,f,h).

On the largest landslides, multiple nested scarps are encountered (g) (Fig. 3D, E, F) that delineate pretty well the displacement transitions within the landslide (for instance b, c), highlighting the variability of the subsurface landslide geometry. All the observations show that the superficial parts of these landslides are extensively fractured, made of non cohesive material and permitting large fluid circulation.

### Monitoring of the landslide displacement

**Figure 4:**
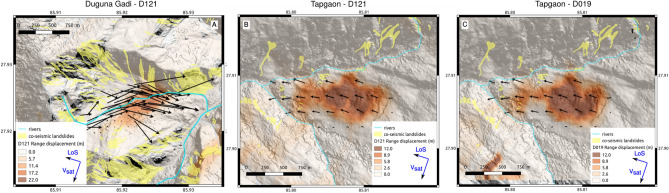
SAR Range displacements from Sentinel-1 images on the Duguna Gadi (**A**) and Tapgaon (**B**,** C**) landslides overlayed over the DEM produced with the December 2015 Pléiades images (see section Methods). The displacements are calculated over the entire 2015 year using the methodology presented in the Methods section, on either the D121 Sentinel-1 track (**A**,** B**) or the D019 Sentinel-1 track (**C**). Arrows correspond to the displacement field of the landslides calculated with Pléiades images. The white squares are the locations at which the time-series of displacements are extracted in Fig. 5. QGIS^23^ was used to create this figure.

Pléiades data allows us to monitor the landslide motions over 3.5 years (Figures 3G–I and S5). All these landslides (except Zhangmu) show a strong acceleration during the seismic sequence. These accelerations reach up to $34\,\pm\, 0.6$ m in 8 months (April-December 2015) on the Duguna Gadi landslide, and between $3.5\,\pm \,0.9$ and $12\,\pm \,0.5$ m for the other ones (Table 1). The refinement of the time-series around these 8 months thanks to the SAR image correlation was possible only on two landslides (Duguna Gadi, Tapgaon, see Fig. 4), correctly oriented and sufficiently large to be visible in the SAR image geometry. Only the range component of the SAR image correlation is sufficiently resolved due the smaller size of the SAR images along the range direction (4 m on average for the range component against 15 m on the azimuth direction for Sentinel-1). Therefore, the correlation of SAR images allows the retrieval of only one dimension of the displacement field, in the line of sight of the satellite. The SAR range displacements display a good consistency with the Pléiades displacement fields, showing clearly the active areas on these two landslides (Fig. 4). Some spatial and magnitude differences between these two datasets exist (Fig. 4A) due to both spatial variations of the landslide motion orientation and steepness that create poorly resolved areas in the SAR image correlation, and large size of the SAR correlation window compared to the landslide size that can lead to discrepancies on the landslide borders or inside the landslide mass due to kinematics heterogeneities^33^.

**Figure 5:**
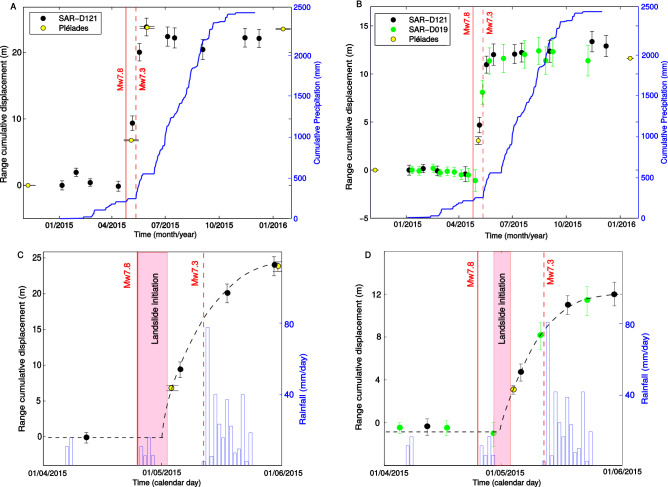
Time-series of landslide displacements along the SAR range direction for Duguna Gadi (**A**,** C**) and Tapgaon landslides (**B**,** D**) from both Pléiades (yellow dots) and SAR (black and green) image correlations at the locations defined in Fig. 4. The blue curve shows the cumulative rainfall as measured by the rain gauge of Bahrabise (see location on map of the Fig. 2) with a daily frequency. The panels (**C**) and (**D**) show a zoom on the two months around the Gorkha earthquake for the two same landslides. The red zone highlights the possible initiation time for the landslide motion. A logarithmic function is also fitted to the post-seismic motions to better estimate this initiation time (dashed black curve).

The time-series (Figs. 5 and S3) show a landslide stability before the earthquake, followed by an acceleration initiating during or few days after the main shock, and a progressive deceleration lasting about 2 months, up to a stability reached in July 2015. Interestingly, on the Tapgaon landslide (Fig. 5B), the time-series clearly display a delay of at least 4 days between the earthquake and the initiation of the acceleration, constrained by a SAR image acquisition on the 29/04/2015. On the Duguna Gadi, no image allows us to detect this delay (Fig. 5A), but the succession of measurements is well fitted by a log curve with an initiation few days after the earthquake. On all the other landslides, where the motion is resolved only with sparse Pléiades images, it is not possible to quantify if the acceleration is co-seismic or is delayed, but it is possible to see that the post-seismic motion is much greater than the co-seismic one (if any).

In the following monsoons, the landslides display a variety of behaviours (Figs. 3 and S5, S6 and S7). Putting aside two landslides (i, j) for which the motion in 2016 or 2017 can not be evaluated due to a lack of Pléiades data, the most common behaviour is the stability (within the uncertainty of the Pléiades measurements) after December 2015 (for landslides b, c, e, h Fig. 3). Such stability is also observed for the landslide (g) based on additional correlation of medium resolution Landsat8 optical images (see Supplementary Materials). Two landslides (d and f) have a continued movement after 2016. For one landslide (a), the motion in the period January 2016-December 2017 is even much larger (10 m) than during the earthquake sequence (<2 m). Besides high anthropogenic disturbance due to road reconstruction, this landslide, connected to the Bhote Koshi river in the upper part of the valley, has been strongly affected by the glacial lake outburst flood (GLOF) from July 2016^34^. So this larger motion might certainly reveal the flooding impact on the landslide destabilization, through erosion of the river banks and landslide toe.

## Discussion

### Common behaviour of the moving landslides

#### A subdued or inexistant response to monsoon and Dolakha earthquake forcings

All landslides, except maybe Zhangmu (a), responded to the earthquake solicitations, with an acceleration that lasted at least several months and not limited to the time of the earthquake. This is a fairly common behaviour for slow-moving landslides as pointed out previously (e.g. Fig. 1). The main novel aspect of this study is given by the time-series of displacement on the Tapgaon and Duguna Gadi landslides, that quantify for the first time the co- and post-seismic motions of landslides in the near field of a large earthquake. First of all, these time-series show that individual rainfalls and monsoons do not impact the motions of these two large landslides. Indeed no motion is observed during the 2015 monsoon (June–October). This is a surprising result given that the combination of earthquake and subsequent rainfalls is usually found to have a larger effect than rainfalls or earthquakes only^7,11^.

Furthermore, the shape of the post-seismic motion (a constant deceleration over 2–3 months, as highlighted by the logarithmic curve from Fig. 5C and D, and no co-seismic step-like acceleration for the Gorkha and Dolakha earthquakes) suggests that the landslide responded to only one transient solicitation. The motion initiation and the highest velocity occur before the Mw 7.3 aftershock, suggesting that the Dolakha earthquake has no effect on the landslide motion. Previous observations on the triggering of rapid landslide also suggest the much lower impact of the Dolakha than the Gorkha earthquake (only 213 among the 15551 landslides mapped by^20^ were triggered by the Dolakha earthquake). The simulated seismic ground motion for the two earthquakes clearly shows a lower shaking for the Dolakha earthquake than for the Gorkha earthquake, despite its proximity to the study area. For instance the recorded highest Peak Ground Acceleration (PGA) close by our area reaches 1.3 g for Gorkha (Chautara station), and around 0.3 g for the Dolakha earthquake in our area of study (USGS shakemap, https://earthquake.usgs.gov/earthquakes/eventpage/us20002926/shakemap). This difference in ground motions can explain, on a first order, the lower effect of the Dolakha earthquake on the landslide kinematics.

#### Co- and post-seismic motions

The landslide co-seismic motions caused by the Gorkha earthquake can not be directly estimated for the slow-moving landslides, where refinements of the time-series with SAR images were not possible. On these landslides the co-seismic motion is much less than the post-seismic motion (Fig. S5), but their exact values are difficult to estimate due to the low acquisition frequency of the Pléiades satellite images. On the landslides clearly not affected by monsoons (b, c, e, g, h), we can however estimate the co-seismic motion to a first order, by extrapolating the post-seismic motion at the time of the earthquake, based on a fit of the post-seismic values with a logarithmic function (Fig. 5C and D). The estimations provide a co-seismic motion of less than 1 m for all the landslides.

**Figure 6:**
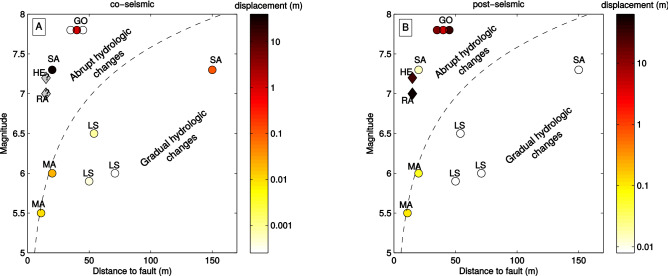
Co- (**A**) and post-seismic (**B**) motions of landslides as a function of the earthquake magnitude and distance to faults, for all the case-studies from the litterature. LS: La Sorbella^10^, MA: Maca^5,11^, SA=Sarpol-Zahab^13^, GO: Gorkha (this study), RA: Racha^4^, HE: Hebgen Lake^12^. The main characteristics of these landslides are exposed in Table S2. The circles denote case-studies with physical measurements of the displacement, whereas diamonds denote the case-studies where only visual testimonies were available. For these latter cases, the co-seismic motion is uncertain and noted with a gray color. The dashed black line represents the limit defined by^18^ to differentiate the abrupt and gradual co-seismic hydrological responses of water-level in wells.

We reported the magnitudes of the co- and post-seismic motions in a comparative graph (Fig. 6), gathering all the existing studies where time-series of slow-moving landslides accelerated by earthquakes exist^5,10,11,13^. We also complement these physical observations with human testimonies of slow-moving landslides, accelerated by earthquakes^4,12^ with time-delays, enabling the separation of the co- and the post-seismic motion. We see (Fig. 6) that the post-seismic effect is vanishing more rapidly with distance/magnitude than the co-seismic effect, highlighting the different processes at the origin of the co- and post-seismic landslide motions, as also pointed out by the analysis of the different landslide displacement time-series presented on Fig. 1. We notice that the magnitude of the post-seismic motion is not function of the landslide slopes, with similar motions on the steep Nepalese landslides than on the very gentle slope of Kirkwood or Chordi- Zhashkva earthflows (Table S2). We also notice that post-seismic motions exist only in the hydrological domain characterized by an abrupt and transient water level changes in the near fields of earthquakes^18^ (Fig. 6B). In opposition, no post-seismic motions are observed in the hydrological domain where gradual and sustained water level changes occur in the intermediate fields of earthquakes.

#### A delayed post-seismic response

An interesting observation of our dataset is the time-delay of at least 4 days between the Gorkha earthquake and the Tapgaon landslide initiation (Fig. 5D), and probably a similar time-delay on the Duguna Gadi landslide (Fig. 5C). This late initiation does not coincide with any strong rainfalls or large (> Mw 6.0) aftershocks in the surrounding, clearly showing that the dynamic loading is not the direct reason of the landslide acceleration. The observation of such time-delays has already been reported in the literature for slow-moving landslides, based on eye witnesses. Two earth slides were reactivated 2 to 3 days after the Mw 7.0 Racha earthquake^4^, and the Kirkwood earthflow was reactivated at least 5 days after the Mw 7.2 Hebgen lake earthquake^12^. Our physical measurements therefore confirm the possibility of late slow-moving landslide initiation by few days following earthquakes, only observed by local inhabitant testimonies until now. All the previous authors attributed this late initiation to changes of groundwater circulation due to the seismic shaking, despite the lack of complementary measurements of groundwater levels.

### A motion controlled by excess pore water pressure?

#### Mechanism of the landslide kinematics

All the observations on the different landslides in the Bhote Koshi valley, whatever their types, geometry or geology, show a similar post-seismic behavior including an acceleration, sometimes delayed by few days, and a relaxation of this motion over several weeks. Furthermore, these landslides in their great majority have no movements in the following years, even during monsoons. These observations lead us to find one single generic law to explain this similar behavior, instead of having an individual conceptual model for each different landslide.

No anthropogenic forcing, like road construction, could explain the initiation of this landslide motion in the first few days after the earthquake. The time-dependent behavior, in particular the observed time-delay of large movement initiation after the main shock, suggests either a fluid effect (as for instance a strong supply of groundwater on the sliding surface or a densification that makes the material more prone to brittle failure in response to subsequent elevations of pore water pressure^35,36^) or a time-dependent fracture process of the landslide shear zone (like progressive failure and maturation of the sliding surface^37^). This last hypothesis is hardly plausible due to the sharp increase of velocity at the landslide initiation, and due to the geomorphology of all the detected landslides, that are mostly reactivations of ancient thick slides with many pre-existing signs of instability. This shows indeed that the landslide failure surface should be already well developed before the earthquake.

Another hypothesis could be a co-seismic landslide compression that can lead to an internal landslide deformation and cracking, and then can favour new water pathways, and makes the landslide more sensitive to further rainstorms or monsoons^35,36^. However the little effect of subsequent monsoons and individual rainfalls (Figs. 3 and 5) on the landslide kinematics makes it a low probable hypothesis. Furthermore, the few observations of aquifer levels in Nepal^38,39^ show that the groundwater levels only start to increase end of June or even July, about 1 month after the monsoon initiation. This excludes the cumulative precipitation as the origin of the observed motion, starting at the end of April. The late initiation and the decrease of velocity though time up to a stability reached at the beginning of the 2015 monsoon suggest instead a local and sudden transient increase of pore-water pressure at depth followed by an evacuation of excess fluids from the landslide shear zone.

Many observations show co- or post-seismic variations of groundwater levels in wells^14,40,41^, stream-flow changes following earthquakes^42^, or fluid and $\hbox {CO}_2$ fluxes changes as well as water temperature changes in hot springs^43^. In particular, very high magnitudes of groundwater level changes have been observed in the near-field of large earthquakes^40,41^, that can overpass by far the magnitudes of groundwater variations created by monsoons^42^. Despite a high variability of the magnitudes of groundwater level changes caused by earthquakes, many observations show a characteristic shape of transient groundwater level changes in the near and intermediate fields of large earthquakes, with an abrupt increase, followed by a slow decay of the water level over a few months^18^. In the near-field of earthquakes, the stream-flow discharge reaches a peak with a time-delay of up to several days^42,44^ in down-rivers or valleys, as a result of released groundwaters from consolidation of sediments or permeability increases^16^, and water diffusion in river catchments from elevated areas to lower areas^16^. Due to the strong similarities between the time-series of co-seismic groundwater changes in the near-field and the time-dependent shape of the landslide kinematics (and particularly the delay observed in our data), we conjecture that the landslide kinematics observed here has a similar origin.

This hypothesis is also supported by the above inter-comparison of all the existing case-studies of post-seismic motions of landslides, which suggests that post-seismic motions only take place in the near-fields of earthquakes, where abrupt and transient water level changes occurred (Fig. 6B). This hydrological behaviour has been explained by material consolidation in the near-field^18^. Therefore, our observations and comparison with the existing database of slow-moving landslides accelerated by earthquakes, suggest that abrupt water increase might originate from the material consolidations in the near-field of earthquakes and provoke the landslide post-seismic motion.

Unfortunately, with the exception of a spring that appeared following the earthquake sequence in Kodari area^43^, no groundwater level measurements are available in the vicinity of the Gorkha earthquake main rupture zone, that could be used to substantiate this hypothesis. Some water level changes were observed in the far-field of the Gorkha earthquake^45^, but data is missing in the near-field. Therefore, the origin of this potential excess pore-water pressure remains a main question.

#### Possible origin of the pore-water pressure excess

**Figure 7:**
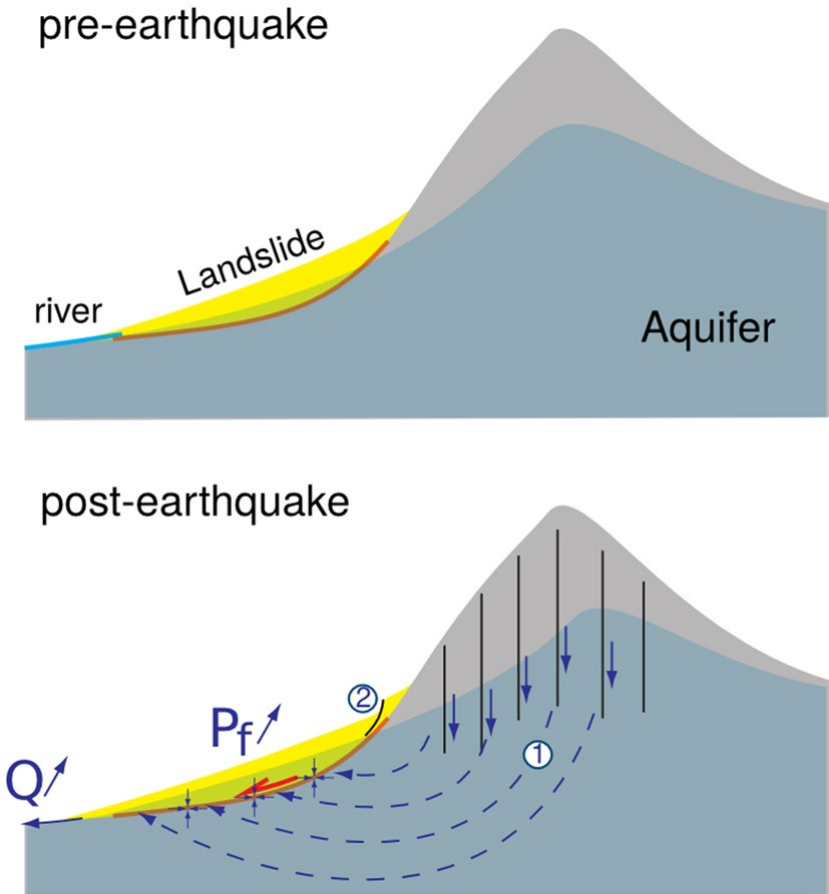
Mechanisms of co-seismic excess pore-water pressure and subsequent transport to the landslide zones (adapted from^16^): (1) Subvertical cracks open during the earthquake, and allow the drainage of water from sediments compaction down to the aquifer, (2) internal landslide reconfiguration, whereby the upslope deformation loads the downslope area, increasing the pore pressure. $P_f$ denotes the fluid pressure at the landslide basal interface, and *Q* denotes the river flow.

In a first scenario, the water would have an external origin, that is coming from the co-seismic material consolidation outside the landslide zone, and be transported to the landslide area either by surface flows or at depth. The Tapgaon and Duguna Gadi landslides, where delayed initiations have been observed, are located inside small valleys, fed by limited contributing catchments of surface areas smaller than 3 km$^2$. This configuration can’t therefore explain the long temporal diffusion (several days) of water-flow excess through water transport in the rivers. However, coseismic damage^11,46^ allows the subvertical draining of water and subsequently the recharge of deep aquifers^16^. The subvertical draining can be caused by the co-seismic shaking and fracturing of the bedrock. This hypothesis is also emphasized by the location of the studied area in the northern part of the Gorkha rupture area, in a zone where co-seismic deformation produced an increase of the North-South tensile stress, favouring an opening of the East-West oriented fractures. The deep aquifers would feed the sources in the lower parts of the massif^16^, where all the observed slow-moving landslides are situated (see Fig. 7). This water diffusion inside the massif can therefore explain the few days time-delays observed here and on other slow-moving landslides^4^.

In a second scenario, the water excess would have an internal origin. This could arise from a reconfiguration of the landslide mass, i.e. the deformation and consolidation of materials in the upper part of the landslide supplies water to downstream parts, resulting in a local increase in pore pressure^47,48^. This process might be revealed by the time-series of displacement at different locations along a longitudinal profile, since water transfer is expected to produce temporally distinct response in the different parts of the landslides (see for instance^49^). On the Tapgaon landslide, whose kinematics is the best resolved in time, all time-series show a synchronous pattern of displacement (Fig. S4), including a similar time-delay of the motion initiation. This synchronicity excludes a priori the landslide reconfiguration as the origin of the pore-water pressure increase. However, this mechanism can’t be fully rejected by our data, because the large size of the correlation windows for SAR images (Fig. S2) prevents to see small blocks at the landslide head-scarp that could have been moving during the earthquake. Indeed, previous studies have shown that even small material supplies or head-scarp retrogressions can modify the hydrology of major landslides^47–49^.

Both of these plausible scenarios would need to be validated by local measurements of groundwater levels and continuous high-frequency landslide displacements during a near-field earthquake, which is a very rare dataset given the few instrumented landslides with continuous measurements in tectonically active zones^48^. Another option to validate these hypotheses would be to iterate similar satellite studies to other case studies, focusing on large landslides in the near field of earthquakes. The growing database of systematic satellite acquisitions and their ever shorter revisit times would make this approach a realistic way to infer the physical processes of landslides during earthquakes.

## Summary and conclusions

Based on 3.5 years of temporally-sparse high-resolution satellite images, we detected 11 slow-moving landslides accelerated by the Gorkha earthquake in April 2015, over 750 km$^2$ in the Bhote Koshi area. Based on field visits, we found that these different landslides are of different types, either thick translational rockslides, or reactivations of very thick paleo-landslides deposits, or mobilization of shallower colluvial debris cover. For the two largest landslides of our database, the use of low-resolution SAR images from Sentinel-1 allowed us to refine the time-series of displacement around the time of the earthquake. These time-series show that these landslides are accelerated by the Gorkha earthquake only, and not by the subsequent Dolakha earthquake neither by the preceeding or following monsoons. These time-series also show that some of the landslides are not accelerated co-seismically but with a time-delay of at least 4 days. The post-seismic motion lasts for 2 months, reaching up to $34\,\pm\, 0.6$ m of displacement, with a progressive deceleration with time up to a stability reached at the very beginning of the monsoon.

The comparison of the inferred co- and post-seismic motions of these landslides with previous observations following other crustal earthquakes shows that post-seismic landslide motions occured at smaller distances than co-seismic ones, and are limited to areas where abrupt and transient water level changes occur. These observations, together with the pattern of movement observed on the two large landslides accelerated by the Gorkha earthquake, strongly suggest that fluids are the cause of the post-seismic motion of landslides at a weekly/monthly scales.

Based on these observations, we investigate several processes to explain the post-seismic motions of landslides, including progressive damage of the landslide material, and hydrological processes linked either to internal reconfigurations of the landslide material, or transfer of fluids from the co-seismic contraction of sediments. Our analysis provided a novel insight into the mechanism of post-seismic landslides at a weekly/monthly scales. We propose that the seismic shaking exfiltrates water from the sediments by contraction and dilation of the material either locally (during the co-seismic reconfiguration of the landslide material) or externally from the landslide area. In this latter case, this excess of water can be transported by subvertical cracks formed during the shaking, feeding a deep aquifer that emerges at the slope toes (Fig. 7), where the slow-moving landslides were detected. We therefore contend that the fluid migration from material consolidation causes the post-seismic motion, through pore-water increase on the shear surface, leading to a potential time lag of several days between the earthquake and the landslide initiation.

This late landslide initiation has also been reported following other major earthquakes, leading sometimes to catastrophic failures^4^, with direct consequences on the hazard associated with earthquake-induced landslides. These new observations therefore emphasize the complex mechanics of landslide triggering by earthquakes: Following the co-seismic phase caused by the dynamic loading, a post-seismic phase of few days or weeks can lead to additional landslides in the near field caused by diffusion of groundwater excess from material contraction. Then, the material damage caused by the shaking, produces a phase of instability of several months to years where rainfalls can easily infiltrates and trigger landslides^7,11^. These different post-seismic phases highlight the hydrologic sensitivity of landslides, showing the need to improve our understanding of the groundwater processes on landslides.

## Methods

### Pléiades image correlation

The data acquisitions are presented in Fig. S1 and Table S1 of the Supplementary Materials. All the Pléiades images are acquired in monoscopic mode, except the December 2015 images, that were acquired in stereo mode.

Time-series of ground displacements over the whole area of study are obtained by a classical scheme^33^ applied on the Pléiades images: (1) DEM construction from the December 2015 stereo acquisitions using Ames Stereo Pipeline v2.5.3 (https://ti.arc.nasa.gov/tech/asr/groups/intelligent-robotics/ngt/stereo/)^24^, (2) Orthorectification of the December 2015 images using the Rational Polynomial Coefficients (RPC), (3) Orthorectification of the other images using the December 2015 DEM and a rigorous sensor model refined from RPC using Ground Control Points (GCPs) extracted from tie-points with the December 2015 orthorectified image using the Cosi-Corr software^50^ (http://www.tectonics.caltech.edu/slip_history/spot_coseis/download_software.html), (4) Image correlation between all pairs of images from the same season (summer or winter) using the Mic-Mac software^51^ (https://micmac.ensg.eu/index.php/Accueil), that is best suited for small objects like landslides. (5) Filtering of the displacement fields with low correlation coefficient values. (6) Time inversion of the displacement fields per pixel using the TIO software (https://sourcesup.renater.fr/www/tio/), separately for the East/West (EW) and North/South (NS) components^33^. This process leads to a time-series of cumulative ground motion displacement along EW and NS. The steps 4, 5 and 6 are applied separately on winter (Nov/Dec 2014 - Dec 2015/Jan 2016 (stereo) - 09/12/2017) and summer (13/06/2014 - 04/05/2015 - 31/05/2015) images, due to large differences of illumination and vegetation that lead to correlations of low quality in between them. A constant bias per pixel in between the summer and winter time-series therefore remains. We fixed this bias by defining a zero-motion during the pre-earthquake time period (13/06/2014 - Nov/Dec 2014 ). This hypothesis is verified a posteriori by comparing with the time-series from SAR images and by checking the consistency of the landslide motion with time (monotonic increase of the cumulative motion).

At each time-step, the uncertainty on the ground motion is calculated with the standard deviation of the ground motion outside the landslide areas. The uncertainties are larger for summer images than winter images, mostly due to a higher vegetation cover. We therefore decided to visually detect slow-moving landslides in the winter displacement fields, by catching patches of motion compatible with slope processes (ground motion oriented in the direction of the slope, where signs of instability are visible on the satellite images). We estimate that all slow-moving landslides larger than 10000 m$^2$ and faster than $\sim$ 1 m/year were detected.

### SAR image correlation

The cloud cover around the time of the earthquake prevents the use of optical images to acquire a high frequency time-series of slow-moving landslide displacements. SAR images, whose signal penetrates clouds, must be used. InSAR measurements is classically used on landslides^29^, but is usually restricted to low displacement rates and for large landslides correctly oriented respect to the SAR orientation. Here, all the detected landslides display displacement rates of several meters in less than 1 year in Pléiades images. These velocities are too high to be detected by C-band InSAR. Therefore, correlation of SAR image amplitudes is used here. Two Sentinel-1 descending tracks (D121 and D019), as well as one ascending track (A085) cover the area of interest. Track A085 is not used here due to the bad geometric configuration of the landslides relatively to the track orientation that greatly limits the visibility of the detected landslides in this track. We limited our analysis with C-band radar to the 2015 period, as the major landslides, large enough to be detected by medium resolution imagery like Sentinel-1, are found to be mostly active at that time and not after (Figs. S6 and S7).

Correlation of SAR image amplitudes has been little applied with Sentinel-1 on landslides^52,53^, which are small objects. Few authors used high-resolution SAR images from TerraSAR-X^54,55^ to derive time-series of landslide displacement. This process is however limited to long-term analysis as the acquisition of data is not systematic. High frequency radar satellites with systematic acquisitions have lower resolutions ($\sim$ 4 × 15 m of pixel size for Sentinel-1), that limits their use for the monitoring of small objects.

Here, we used a correlation tool that implement the classical Normalized Cross-Correlation method adapted and optimized to SAR images^56^. The tool also provides a chain of workflows that co-register and compute the displacements from time series of images (https://efidir.poleterresolide.fr/index.php/effidir-tools). This method allows us to differentiate the size of the master correlation window in range and azimuth, which is key to measure the ground displacement of small objects in radar images^57^. We test different combinations of range and azimuth window sizes. The optimum sizes are found by maximizing the signal to noise ratio of the displacement field obtained by correlating two images encompassing an active slow-moving landslide, previously detected (see Fig. S2 from the Supplementary Material). The correlation window is fixed at 95 pixels in range and 73 in azimuth.

The images of the same track are all correlated two by two, and inverted using a time-inversion^33^ using the TIO algorithm (https://sourcesup.renater.fr/www/tio/). A weight between pairs, function of their time-separations is used in the inversion process. This process leads to a time-series of ground displacement for the two tracks D121 and D019. Uncertainty of these time-series are estimated by the standard deviation of the ground motion outside the landslide areas.

## Supplementary Information

Supplementary Information 1.

## Data Availability

SLC data from Sentinel-1 is publicly available on the PEPS website (https://peps.cnes.fr/rocket/). Pléiades images are available upon request to AIRBUS DS https://www.intelligence-airbusds.com/geostore/. The datasets of the different image processing (displacement fields, DEM) are available upon request to the authors. The rainfall data from rain gauges is available upon request to the Department of Hydrology and Meteorology of Nepal (http://dhm.gov.np/requestfordata/).
