# Supplementary material for: SAR and optical images correlation illuminates post-seismic landslide motion after the Mw 7.8 Gorkha earthquake (Nepal)
Source: Sci Rep. 2022 Apr 15;12:6266. doi: 10.1038/s41598-022-10016-2 (PMC9012803; doi:10.1038/s41598-022-10016-2)
Supplement: Supplementary file 1 — Supplementary Information 1. [file 41598_2022_10016_MOESM1_ESM.pdf]

Supplementary Information for the paper "SAR and optical images correlation illuminates post-seismic landslide motion after the Mw7.8 Gorkha earthquake (Nepal)"

Pascal Lacroix[1,2,*], **Theo Gavillon**[1], **Clément Bouchant**[1], **Jerome Lavé**[3], **Jean-Louis Mugnier**[1], **Samir Dhungel**[4], **and Flavien Vernier**[5]

[1]ISTerre, University Grenoble Alpes, University Savoie Mont-Blanc, CNRS, IRD, 38000 Grenoble, France
[2]The Njord Centre, Department of Geosciences, University of Oslo, Oslo, Norway
[3]Centre de Recherches Petrographiques et Geochimiques, CNRS, 54501 Vandoeuvre-les-Nancy, France
[4]Tribhuvan University, 44618 Kirtipur, Nepal
[5]LISTIC, University Savoie Mont-Blanc, Annecy, France.
[*]pascal.lacroix@univ-grenoble-alpes.fr

Contents of this file

1 Description of the data used to derive time-series of ground displacement

2 Sensitivity analysis of the SAR correlation to window sizes

3 Time-series of landslide displacement from SAR image correlation

4 Zoomed maps and individual displacement time-series obtained from Pleiades data

5 2014-2018 Landsat-8 image correlation

6 Characteristics of the slow-moving landslides mentioned in the text

7 Rainfall data from rain gauges

1 Satellite data

Table S1. Satellite acquisition dates

Satellite - Track	Dates
Sentinel-1 D121	06/01/2015 30/01/2015 23/02/2015 12/04/2015 06/05/2015 18/05/2015 30/05/2015 05/07/2015 17/07/2015 03/09/2015 14/11/2015 08/12/2015
Sentinel-1 D019	11/01/2015 23/01/2015 16/02/2015 28/02/2015 12/03/2015 24/03/2015 05/04/2015 17/04/2015 29/04/2015 11/05/2015 23/05/2015 16/06/2015 22/07/2015 15/08/2015 27/08/2015 08/09/2015 07/11/2015
Pléiades	13/06/2014 - Nov/Dec 2014 - 04/05/2015 - 31/05/2015 - Dec 2015/Jan 2016 (stereo) - 09/12/2017

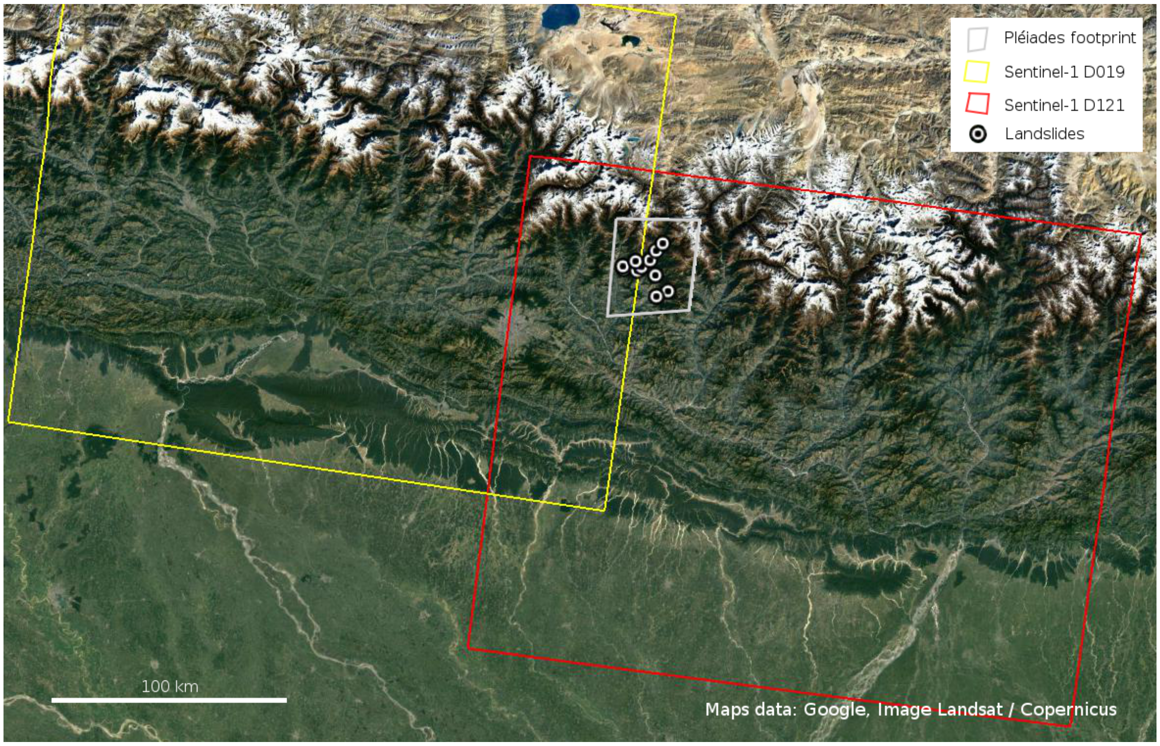

Figure S1. Footprints of the satellite images used in this study, both Sentinel-1 data and Pléiades images, overlayed over a Google-Earth view https://earth.google.com/ of the area.
.

2 SAR correlation parameters

The SAR image correlations was realized using the Normalized Cross-Correlation (NCC) method adapted and optimized to SAR images[1]. This method allows us to differentiate the size of the master correlation window in range (W_{Ra}) and azimuth (W_{Az}). To find the optimum parameters for the correlation, we tried different combinations (W_{Ra}, W_{Az}) on an area affected by a slow-moving landslide (Figure S2), and optimized the ratio between the mean signal on the landslide and the noise outside the landslide (estimated by the standard deviation of the measurements outside the landslide). We found the optimum window size to be 95 pixels in range and 73 in azimuth (Figure S2C).

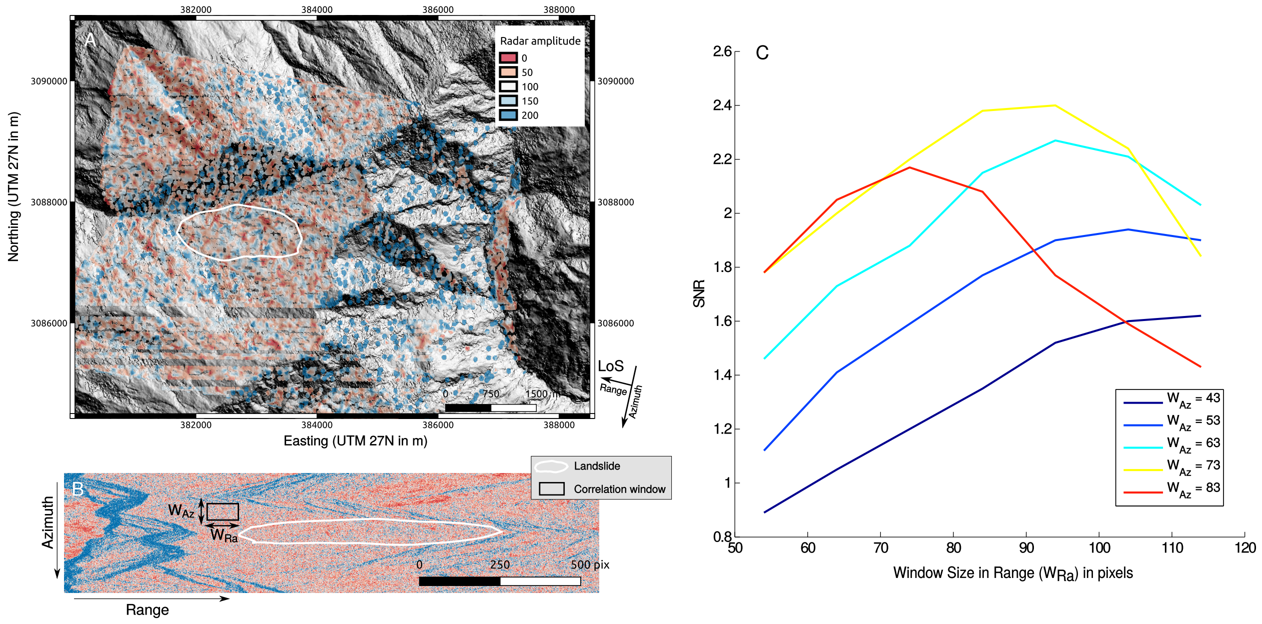

Figure S2. Radar image of Sentinel-1 downloaded from the PEPS server (https://peps.cnes.fr/rocket/) on the Tapgaon landslide from the descending orbit D019 as seen projected onto the ground and overlayed over the DEM created with Ames Stereo Pipeline v2.5.3 (https://ti.arc.nasa.gov/tech/asr/groups/intelligent-robotics/ngt/stereo/) applied on the Pléiades images from December 2015 (A), or in the radar geometry (B). The landslide is contoured with a thick white line. Panel C shows the results of the SNR (defined as the ratio between the mean displacement on the landslide and the standard deviation of the displacement outside the landslide, measured between two pre/post- seismic radar images.) for different sizes of the correlation window in Range (W_{Ra}) and in Azimuth (W_{Az}). QGIS[2] was used to create this figure.

3 Time-series of landslide displacement from SAR image correlation

We present the whole time-series of ground displacement over the Tapgaon landslide based on the correlation and inversion of SAR images. This time-series is represented for the whole area in Figure S3 and for 3 selected points on the landslide in Figure S4.

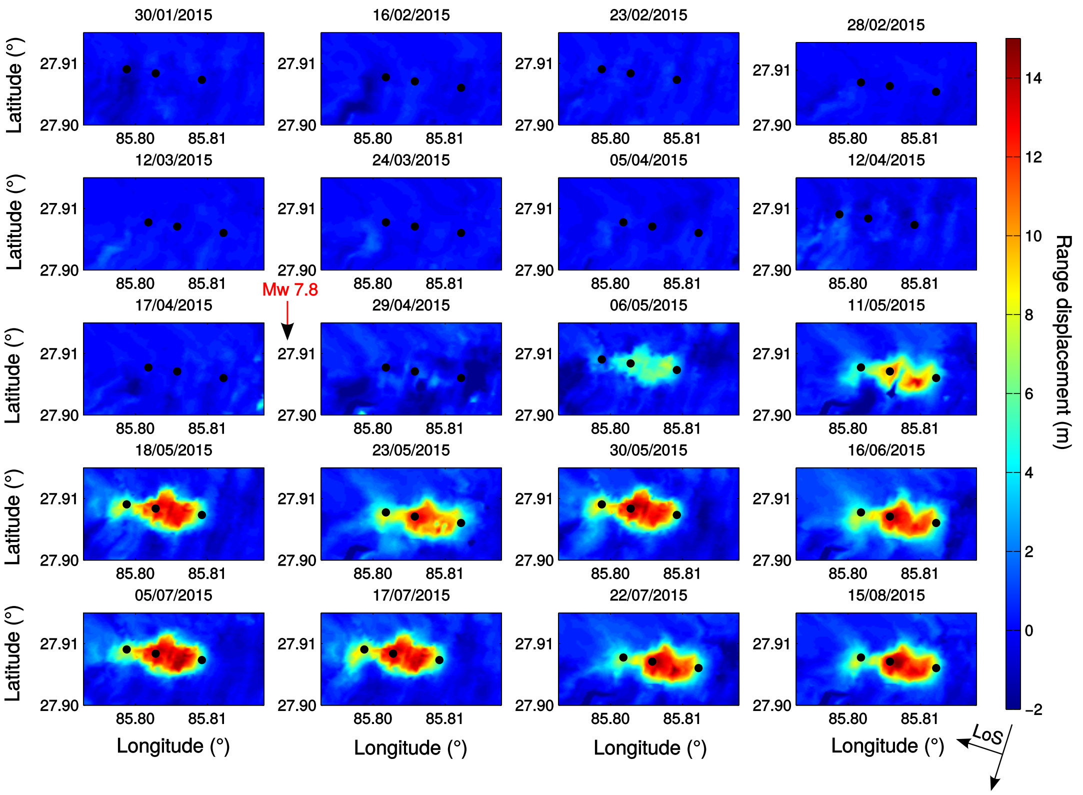

Figure S3. Time-series of ground displacement (along the Range direction) calculated by correlation of Sentinel-1 images from the two descending tracks D019 and D121, over the Tapgaon landslide. The red arrow indicates the time of the Mw7.8 earthquake.

.

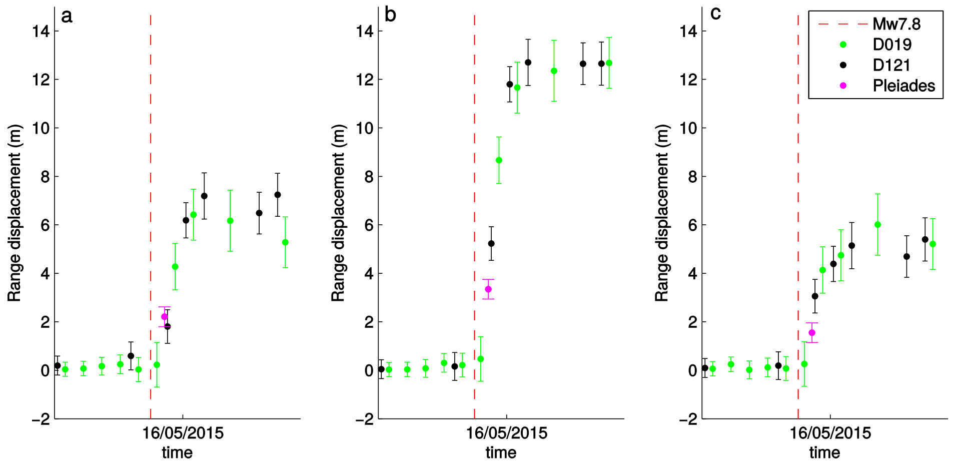

Figure S4. Displacement time-series of the three black points shown in Figure S3 corresponding at 3 location of the landslide: bottom part (a), middle part (b), upper part (c).

.

4 Zooms of Pléiades displacement fields on the slow-moving landslides

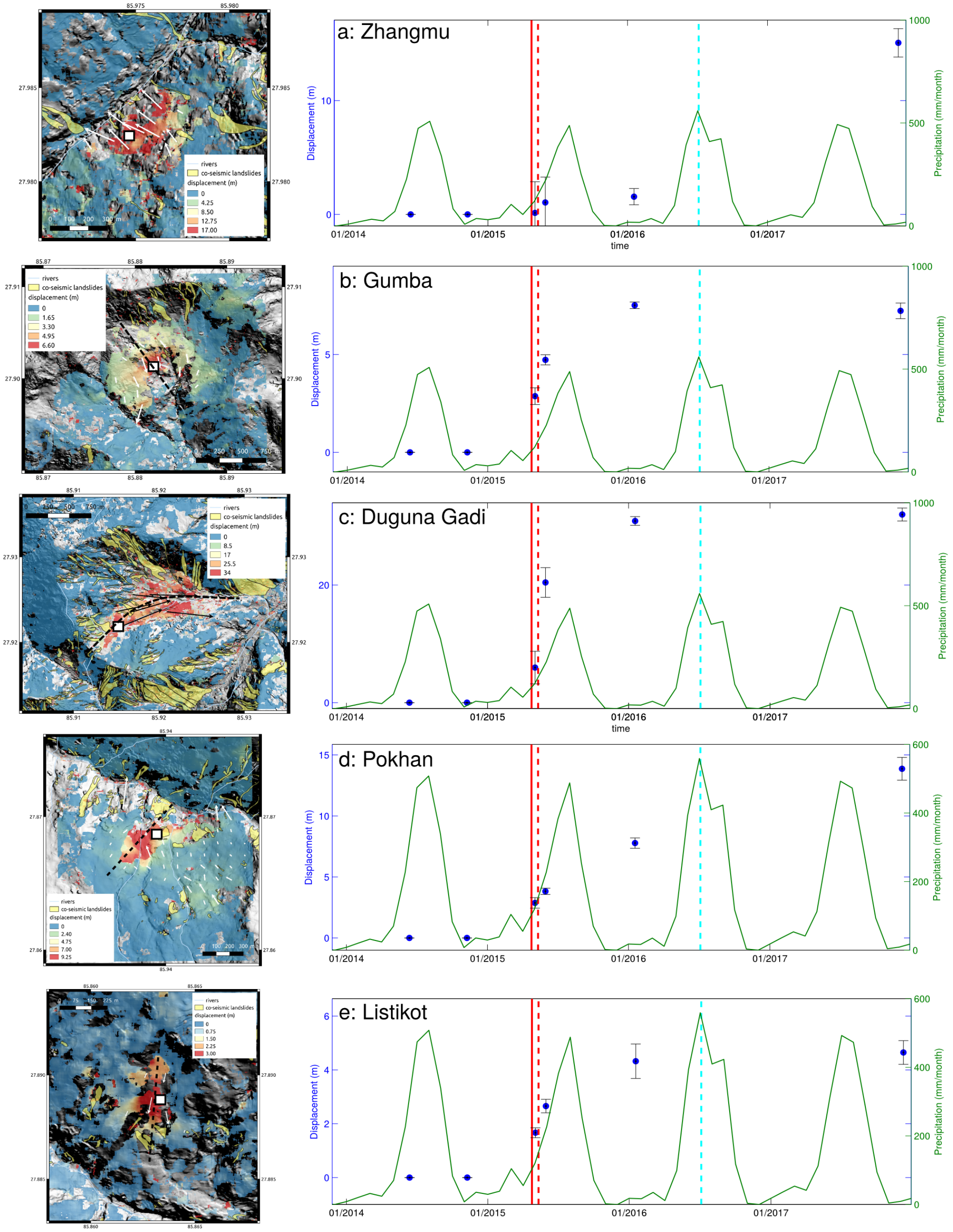
a: Zhangmu
b: Gumba
c: Duguna Gadi
d: Pokhan
e: Listikot
Displacement (m)
Precipitation (mm/month)
01/2014
01/2015
01/2016
01/2017
time
rivers
co-seismic landslides
displacement (m)

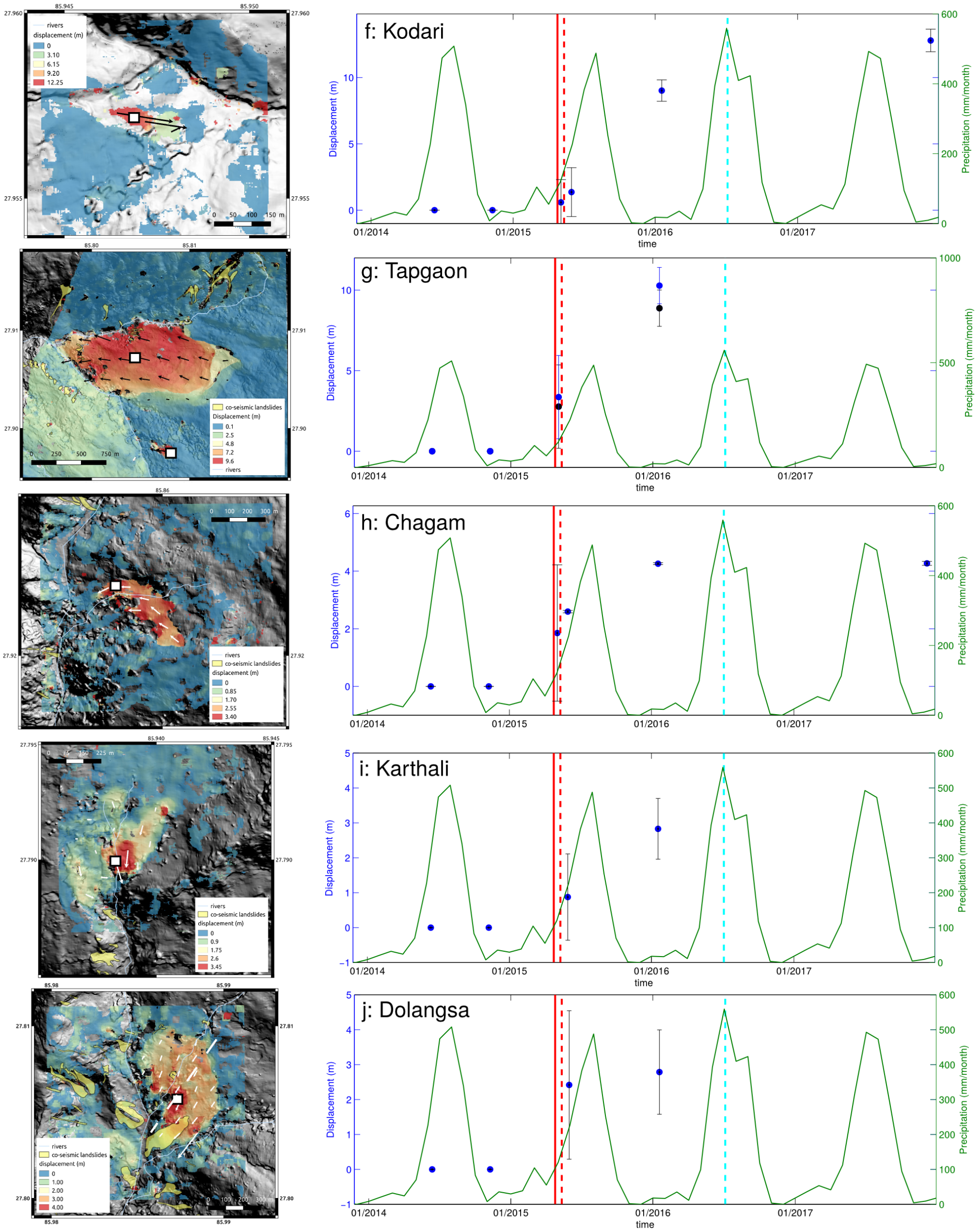

Figure S5. Zooms on the detected slow-moving landslides. From top to bottom: (a) Zhangmu ,(b) Gumba ,(c) Duguna Gadi, (d) Pokhan, (e) Listikot, (f) Kodari, (g)Tapgaon, (h) Chagam, (i) Karthali, (j) Dolangsa. On the left, total horizontal displacement field over the entire available time-period obtained with the Pléiades images calculated using the methodology presented in the main text. On the right, time-series of cumulative displacement obtained with Pl'eiades images of one point situated on the most active area of each landslide, located on the leftern map with a white square. The Gorkha and Dolakha earthquake timings are represented with plain and dashed vertical red lines respectively. The timing of the Glacial Lake Outburst Flood (GLOF) in the Bhote Koshi valley[3] is represented with a dashed vertical cyan line. QGIS[2], matlab (www.mathworks.com) and Inkscape (www.inkscape.org) were used to create this figure.

5 2014-2018 Landsat-8 image correlation

The Pléiades data do not cover the whole 2014-2018 period for all the landslides, so that the relative part of the landslide motion during the 2015 period and during the following monsoons can't be assessed for 3 of the landslides (Tapgaon, Karthali, Dolangsa). To overcome partly this limitation, we compute the displacement fields of the largest landslides (Duguna Gadi and Tapgaon), that can be detected by medium resolution optical satellites like Landsat-8, with a resolution of 15m[4]. We chose cloud-free images from similar periods in the different years (October-December), to decrease artifacts from shadows[4]. The Landsat-8 images were downloaded from the earthexplorer server (https://earthexplorer.usgs.gov/). We correlate the Landsat-8 images over two periods: 23/12/2014-07/10/2015 (that includes the Gorkha earthquake and the 2015 monsoon), 07/10-2015-31/10/2018 (that includes 3 successive monsoons). The correlation is done with the software cosi-corr[5] in the frequency domain with a multi-scale sliding window (64 by 32 pixels). These data were chosen following the work realized with similar data on another landslide area[4].

The landslide motions are similar in patterns and in magnitudes (Figures S6 and S7) compared to what has been calculated with Pléiades images (Figure S5. The motion is clearly concentrated during the 2015 period, and no motion can be detected after, showing the little effect of the following monsoons compared to the co- and post-seismic phase.

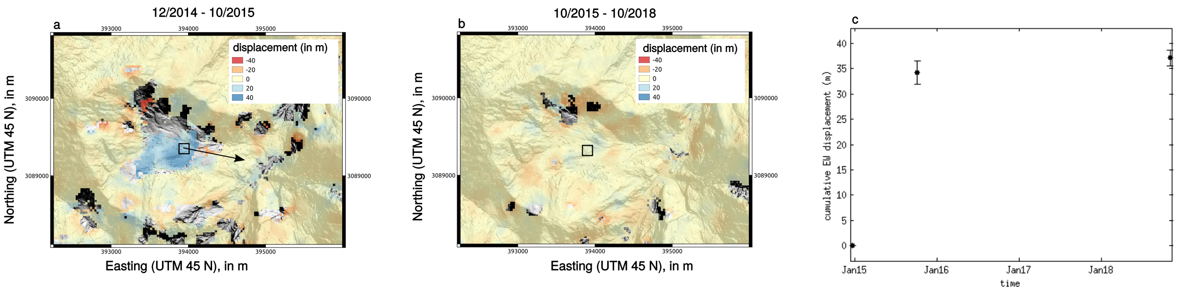

Figure S6. EW displacement fields of the Duguna Gadi landslide over the period 23/12/2014-07/10/2015 (a) 07/10/2015-31/10/2018 (b) calculated with the Cosi-Corr software[5] applied on the Landsat-8 data (see the text from section 5). Positive values correspond to displacement toward the East. The time-series of cumulative displacement for the point on the landslide marked with a black square is represented on the panel c. QGIS[2] was used to create this figure.

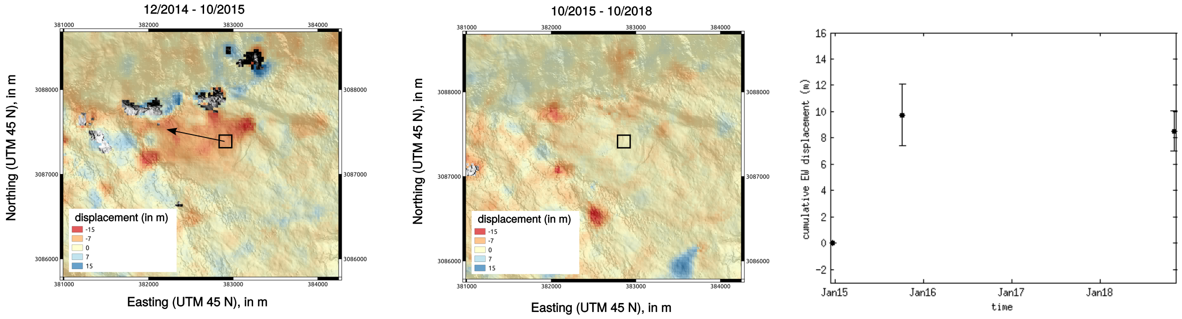

Figure S7. EW displacement fields of the Tapgaon landslide over the period 23/12/2014-07/10/2015 (a) 07/10/2015-31/10/2018 (b) calculated with the Cosi-Corr software[5] applied on the Landsat-8 data (see the text from section 5). Positive values correspond to displacement toward the East. The time-series of cumulative displacement for the point on the landslide marked with a black square is represented on the panel c. QGIS[2] was used to create this figure.

6 Characteristics of the slow-moving landslides mentioned in the text

The following Table summarizes the main characteristics of the slow-moving landslides that have been reported by previous studies to accelerate in a co- or/and a post-seismic manner. The co- and post-seismic kinematics of these landslides are shown in the Figure 6 of the main text.

Table S2. Characteristics of the slow-moving landslides previously reported to have been accelerated by earthquakes in a co- or/and post-seismic manner.

Landslide name	mean slope	max thickness	mechanism	geology
La Sorbella[6]	8°	35m	Earth slide	clay and silt
Maca[7]	11°	80m	Lateral spreading	Quaternary fluvio- lacustrine and rock avalanche deposits
Sarpol-Zahab landslides (Mehr, Mela-Kabod...)[8]	$8\text{-}14^o$	30-300m	Rock slide	limestone blocks sliding over a shale layer
Racha landslides (Zhashkva, Chordi)[9]	$5\text{-}10^o$	30-60	Earth slide	claystone of the Maikop Formation
Hebgen Lake (Kirkwood)[10]	10°	?	Earth flow	Clayed soil and colluvium
Bhote Koshi landslides (Tapgaon, Duguna Gadi. . . .), (this study)	$17\text{-}32^o$	?	Translational rockslides or reactivations of very thick paleo-landslides deposits or mobilization of shallow colluvial debris cover	debris or colluvial cover, fractured-damaged bedrock

7 Rainfall data from rain gauges

Two weather stations, measuring the daily precipitations, maintained manually by the Department of Hydrology and Meterology of Nepal (DHM) were available in our area of study in 2015: one in Dhumthang, one in Bahrabise separated by about 10 km (see map from figure S8). Both weather stations have missing data for short periods: the Bahrabise station in February and March 2015, the Dhumthang station in June and July during the monsoon. We therefore rely more on the data from the Bahrabise station, where missing data are from the drier season. Both stations present very similar amount of daily rainfall in the days where no gaps exists. Therefore we decided to fill these gaps with the available data from the other station (Figure S8). In the main text the newly computed rainfall time-series from the Bahrabise station is shown.

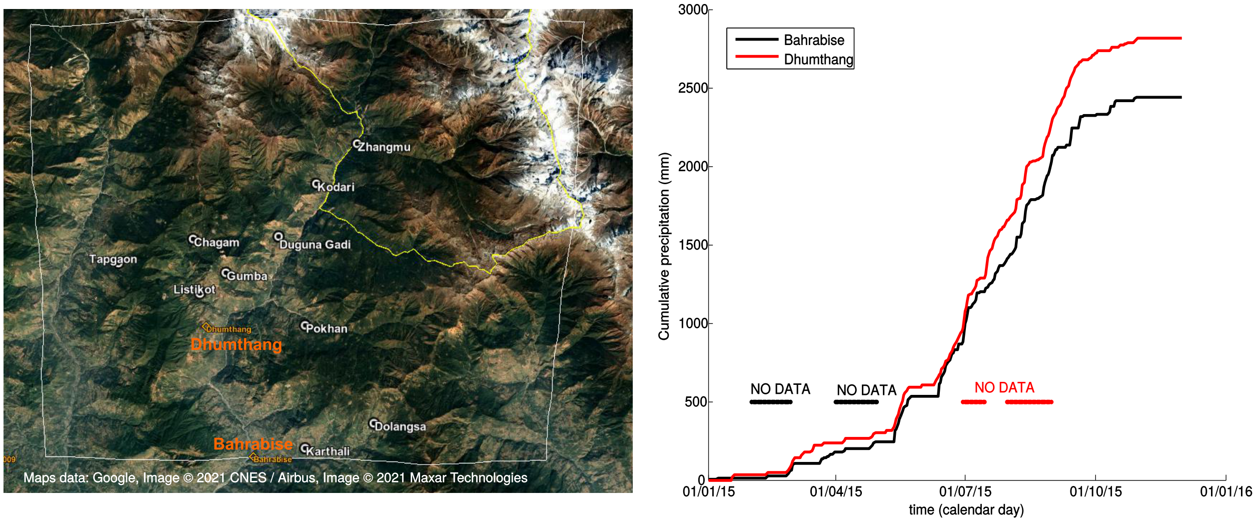

Figure S8. Google-Earth view of the area (left) including the two rain gauges recording rainfalls on a daily basis. The time series of cumulative rainfall is shown (right) for these two stations for the year 2015. Google-Earth https://earth.google.com/ was used to create this Figure.

.

References

1. Vernier, F. *et al.* Fast correlation technique for glacier flow monitoring by digital camera and space-borne sar images. *EURASIP J. on Image Video Process.* **2011**, 1–15 (2011).
2. QGIS Development Team. *QGIS Geographic Information System.* QGIS Association (2021).
3. Cook, K. L., Andermann, C., Gimbert, F., Adhikari, B. R. & Hovius, N. Glacial lake outburst floods as drivers of fluvial erosion in the himalaya. *Science* **362**, 53–57 (2018).
4. Lacroix, P., Araujo, G., Hollingsworth, J. & Taipe, E. Self-entrainment motion of a slow-moving landslide inferred from landsat-8 time series. *J. Geophys. Res. Earth Surf.* **124**, 1201–1216 (2019).
5. Leprince, S., Barbot, S., Ayoub, F. & Avouac, J.-P. Automatic and Precise Orthorectification, Coregistration, and Subpixel Correlation of Satellite Images, Application to Ground Deformation Measurements. *IEEE Transactions on Geosci. Remote. Sens.* **45**, 1529–1558, DOI: 10.1109/TGRS.2006.888937 (2007).
6. Ruggeri, P., Fruzzetti, V. M., Ferretti, A. & Scarpelli, G. Seismic and rainfall induced displacements of an existing landslide: Findings from the continuous monitoring. *Geosciences* **10**, 90 (2020).
7. Zerathe, S. *et al.* Morphology, structure and kinematics of a rainfall controlled slow-moving Andean landslide, Peru: The Maca slow-moving Andean landslide. *Earth Surf. Process. Landforms* **41**, 1477–1493, DOI: 10.1002/esp.3913 (2016).
8. Cheaib, A. *et al.* Landslides induced by the 2017 mw7.3 sarpol-zahab earthquake (iran). *Landslides* (accepted).
9. Jibson, R. W., Prentice, C. S., Borissoff, B. A., Rogozhin, E. A. & Langer, C. J. Some observations of landslides triggered by the 29 April 1991 Racha earthquake, Republic of Georgia. *Bull. Seismol. Soc. Am.* **84**, 963–973 (1994).
10. Hadley, J. Landslides and related phenomena accompanying the hebgen lake earthquake of august 17, 1959. *US Geol. Surv. Prof. Pap.* **435**, 107–138 (1964).
